# Inverse expression of somatostatin and CXCR4 chemokine receptors in gastroenteropancreatic neuroendocrine neoplasms of different malignancy

**DOI:** 10.18632/oncotarget.4491

**Published:** 2015-07-14

**Authors:** Daniel Kaemmerer, Tina Träger, Maike Hoffmeister, Bence Sipos, Merten Hommann, Järg Sänger, Stefan Schulz, Amelie Lupp

**Affiliations:** ^1^ Department of General and Visceral Surgery, Zentralklinik Bad Berka, Bad Berka, Germany; ^2^ Department of Pharmacology and Toxicology, Jena University Hospital, Friedrich Schiller University, Jena, Germany; ^3^ Institute of Pathology, University Hospital Tuebingen, Germany; ^4^ Institute of Pathology and Cytology, Bad Berka, Germany

**Keywords:** somatostatin receptor, neuroendocrine tumor, neuroendocrine carcinoma, chemokine receptor, CXCR4

## Abstract

**Introduction:**

Somatostatin receptors (SSTR) are widely distributed in well-differentiated neuroendocrine neoplasms (NEN) and serve as primary targets for diagnostics and treatment. An overexpression of the chemokine receptor CXCR4, in contrast, is considered to be present mainly in highly proliferative and advanced tumors. Comparative data are still lacking, however, for neuroendocrine carcinomas (NEC).

**Methods:**

SSTR subtype (1, 2A, 3, 5) and CXCR4 expression was evaluated in G1 (*n* = 31), G2 (*n* = 47), and low (G3a; Ki-67: 21–49%; *n* = 21) and highly proliferative (G3b; Ki-67: >50%, *n* = 22) G3 (total *n* = 43) gastroenteropancreatic NEN samples by performing immunohistochemistry with monoclonal rabbit anti-human anti-SSTR and anti-CXCR4 antibodies, respectively, and was correlated with clinical data.

**Results:**

Both CXCR4 and SSTR were widely expressed in all tumors investigated. CXCR4 expression differed significantly between the G1 and G3 specimens and within the G3 group (G3a to G3b), and was positively correlated with Ki-67 expression. SSTR2A, in contrast, exhibited an inverse association with Ki-67. SSTR2A was highly expressed in G1 and G2 tumors, but was significantly less abundant in G3 carcinomas. Additionally, SSTR1 expression was higher in G3a than in G3b tumors.

**Conclusion:**

We observed an elevation in CXCR4 and a decrease in SSTR2A expression with increasing malignancy. Interestingly, 23% of the G3 specimens had strong SSTR2A expression.

Because CXCR4 was strongly expressed in highly proliferative G3 carcinomas, it is an interesting new target and needs to be validated in larger studies.

## INTRODUCTION

Gastroenteropancreatic neuroendocrine neoplasms (GEP-NEN) comprise a heterogeneous group of tumors originating from the endocrine cells of the intestinal tract. Although they are rare, there has been a gradual increase in the incidence of GEP-NEN in recent years, likely due to an improved sensitivity of the imaging techniques employed [1–3]. The WHO (World Health Organization) classification of GEP-NEN separates well-differentiated neuroendocrine tumors (NET) into low-grade (G1) and intermediate grade (G2) categories, and poorly differentiated neuroendocrine carcinomas (NEC) into a high grade (G3) category. Tumor grade is determined, in part, by the Ki-67 proliferation index; G1, G2, and G3 tumors are defined as having a Ki-67 index of <2%, 3–20%, and >20%, respectively [4]. However, newer investigations report biological and histopathological differences within the G3 category [5, 6]. Sorbye et al. reported that a Ki-67 of 55% was the best cut-off value for predicting patient response to platinum-based chemotherapy [7]. Patients with a Ki-67 <55% had a poor response to this therapy, but a longer survival than patients with more proliferative tumors (Ki-67 >55%) [7]. As a result, different types of cytotoxic treatments are recommended [8, 9]. The grading is inversely associated with overall survival (OS). Whereas NEN (G1) have a good prognosis with a 5-year survival rate of 64%, NEC are characterized by a limited 5-year survival rate of less than 12% [10].

More than 80% of NEN express somatostatin receptors (SSTR), mainly the SSTR2A subtype. Moreover, in the PROMID and CLARINET studies, an anti-proliferative response was demonstrated for somatostatin analogs in G1 and G2 NEN [11, 12]. However, the significance of somatostatin analogs for the diagnosis and treatment of G3 carcinomas has not been determined, primarily due to the expected low expression of SSTR and the estimated low anti-proliferative efficacy of these substances.

The chemokine receptor CXCR4 is expressed in more than 23 different tumor types [13]. Furthermore, many studies have shown that increased CXCR4 expression is associated with early metastasis, tumor recurrence, and poor patient outcome. Several CXCR4 antagonists have already been synthesized (e.g., AMD3100 (plerixafor), AMD3465, TF 14016, BMS-936564), which display a high anti-proliferative capacity both *in vitro* and in different animal tumor models [14]. Thus, several clinical studies to evaluate the efficacy of CXCR4 antagonists in cancer patients have been initiated, some of which are still ongoing [15].

Although some studies have shown that CXCR4 is expressed in G3 NEC as well, comprehensive data are still missing for this tumor type [16, 17]. Thus, the present investigation aimed to determine whether different SSTR (SSTR1, 2A, 3 and 5) and CXCR4 are co-expressed in G1-G3 neuroendocrine tumors and carcinomas by means of immunohistochemistry, using highly specific monoclonal antibodies.

## RESULTS

### Primary tumor origin

The majority of the primaries and the metastases investigated in the present investigation (*n* = 121) were derived from the ileum (49/121 ≙ 40%), pancreas (29/121 ≙ 24%), colon/rectum (27/121 ≙ 22%), appendix (5/121 ≙ 4%) and stomach (1/11 ≙ 0.8%). In 10/121 cases (≙ 8%) they were from other origins. All metastases were either liver, lymph node or peritoneal metastases.

### Immunohistochemistry

**Figure 1:**
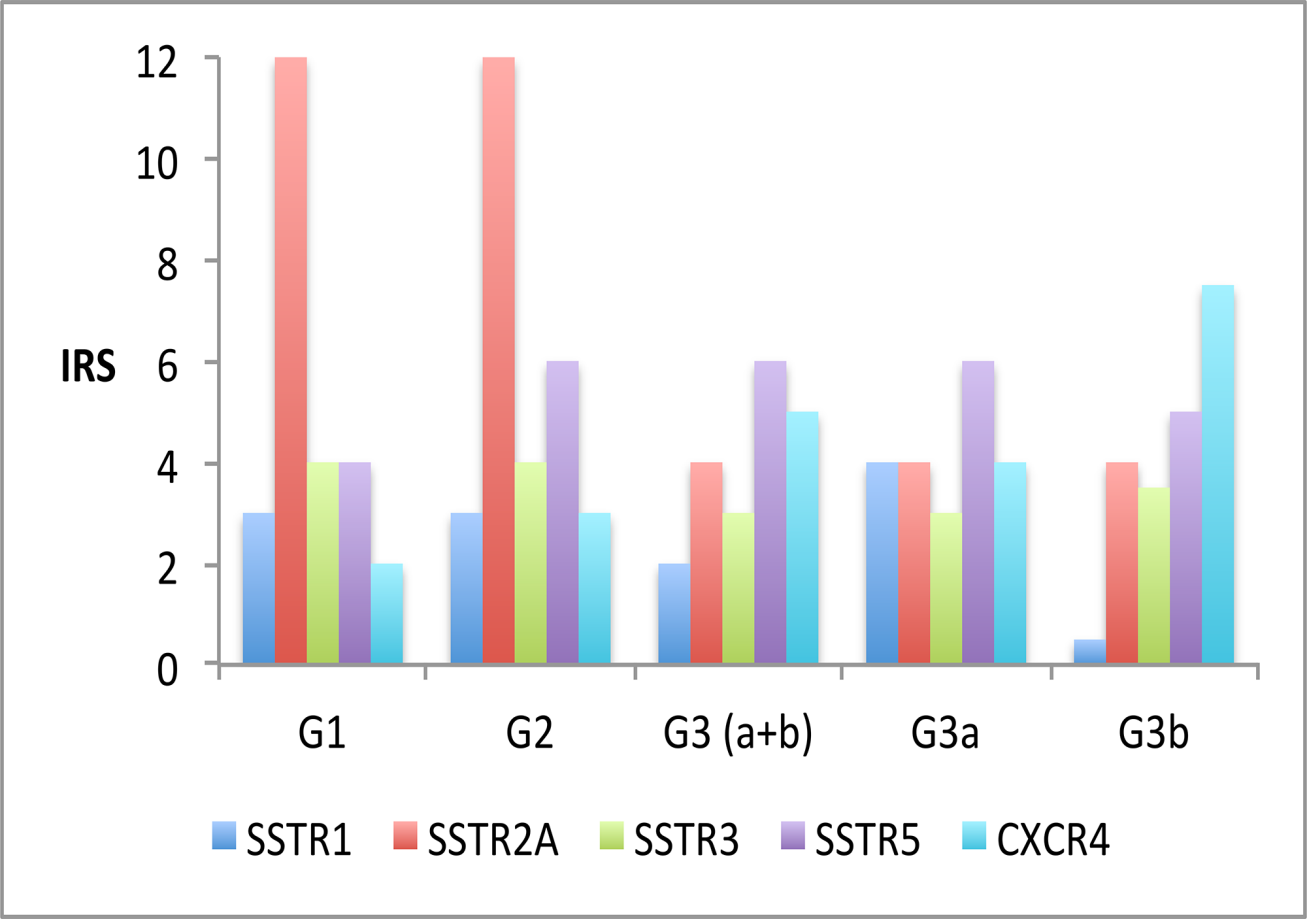
Overview over the SSTR-subtype distribution (median values) within the different tumor groups (G1–G3)

**Figure 2:**
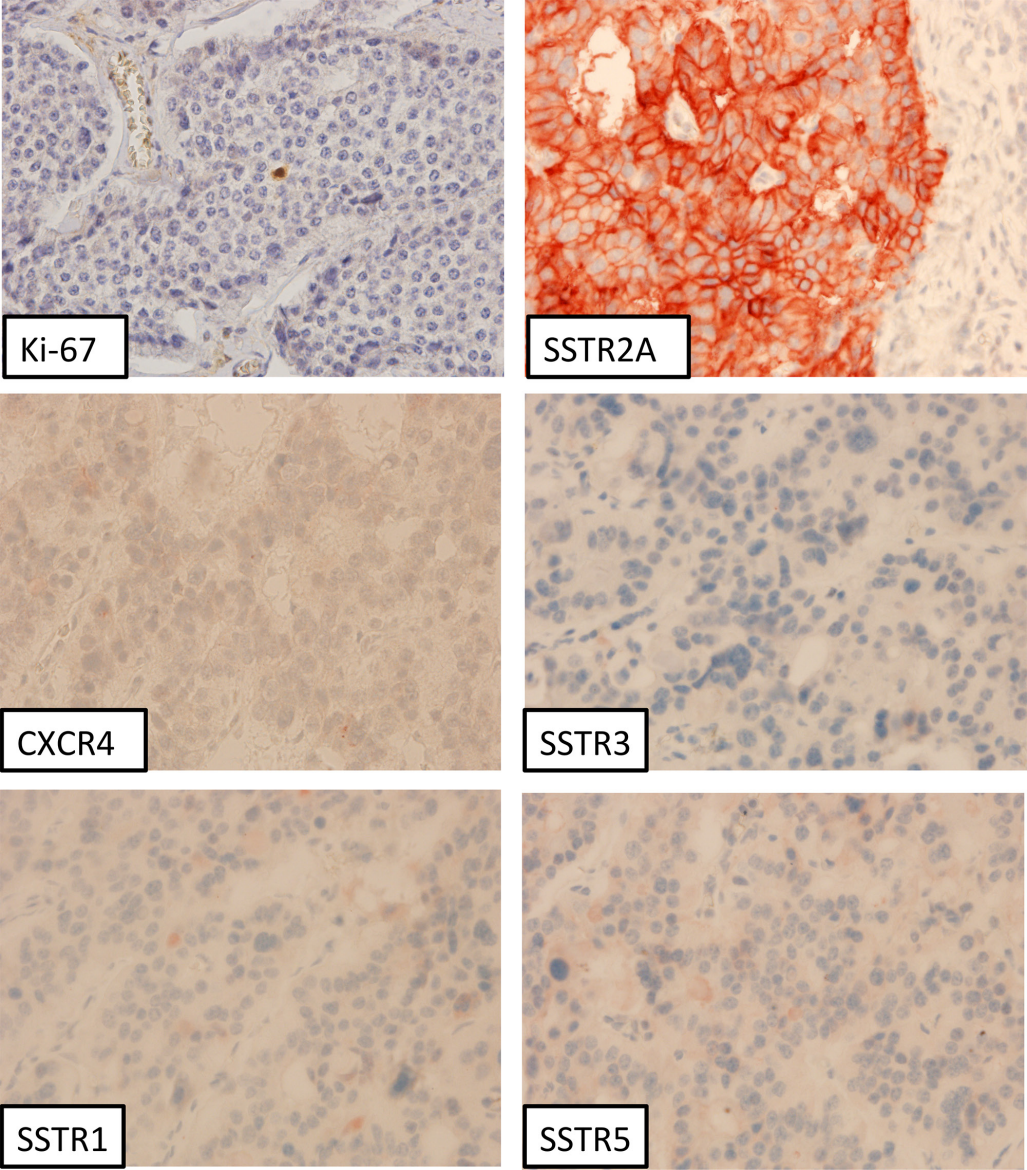
Liver metastasis of a neuroendocrine tumor (G1, Ki-67 <2%); immunohistochemistry, counterstaining with hematoxylin; original magnification: x400

**Figure 3:**
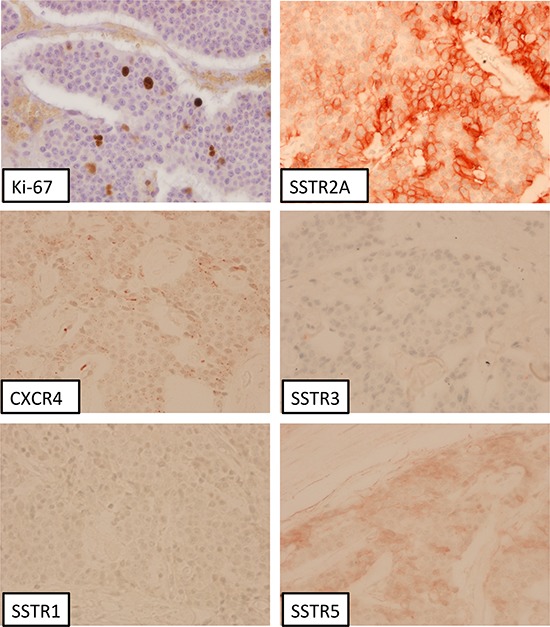
Liver metastasis of a neuroendocrine tumor (G2, Ki-67: 4%); immunohistochemistry, counterstaining with hematoxylin; original magnification: x400

Figure 1 presents an overview about the SSTR subtype distribution within the different tumor groups (median values). Between G1 and G3a tumors a significant difference was observed with respect to the IRS of the SSTR2A (12.0 vs. 4.0; *p* < 0.001) and of the CXCR4 expression (2.0 vs. 4.0; *p* = 0.049) (Table 1). The G1 group differed from the G3b tumors in the IRS of the SSTR1 (3.0 vs. 0.5; *p* = 0.002), the SSTR2A (12.0 vs. 4.0; *p* < 0.001) and the CXCR4 expression (2.0 vs. 7.5; *p* < 0.001). In Figure 2 and Figure 3 photomicrographs of immunohistochemical stainings of a patient with a liver metastasis of a G1 and of a patient with a G2 neuroendocrine tumor are depicted exemplarily.

**Table 1:** Receptor expression data

	G1 *N* = 18	G2 *N* = 22	G3 *N* = 24	
			G3a *N* = 10	G3b *N* = 14
***CXCR4 [IRS]***				
Median	2.0	3.0	4.0	7.5
Mean	2.8	3.4	4.1	7.6
Min	0	0	0	0
Max	8	12	9	12
SD	2.2	2.6	2.3	4.1
***SSTR1 [IRS]***				
Median	3.0	3.0	4.0	0.5
Mean	3.1	3.1	4.1	1.6
Min	0	0	1	0
Max	6	8	8	6
SD	1.7	2.4	2.1	2.0
***SSTR2A [IRS]***				
Median	12	12	4.0	4.0
Mean	10.1	10.0	4.6	5.0
Min	4	0	0	0
Max	12	12	12	12
SD	2.6	3.1	3.4	3.5
***SSTR3 [IRS]***				
Median	4.0	4.0	3.0	3.5
Mean	4.4	4.6	3.3	2.7
Min	1	0	0	0
Max	9	9	8	6
SD	2.0	2.4	2.0	2.1
***SSTR5 [IRS]***				
Median	4.0	6.0	6.0	5.0
Mean	3.9	5.5	5.4	5.4
Min	0	1	0	2
Max	9	12	9	12
SD	2.4	2.5	2.8	2.4

**Figure 4:**
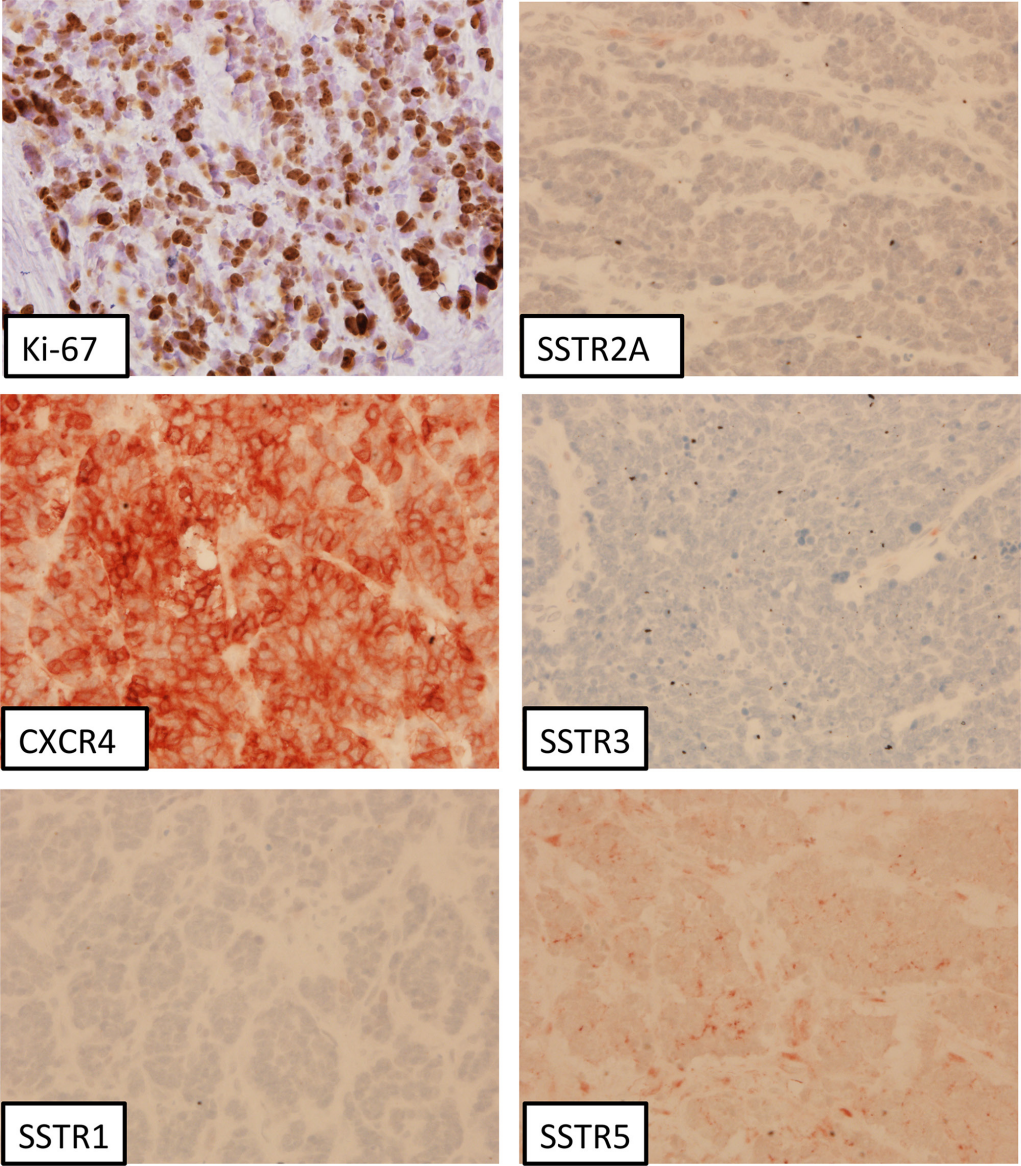
Neuroendocrine carcinoma of the colon ascendens (G3, Ki-67: 80%); immunohistochemistry, counterstaining with hematoxylin; original magnification: x400

G2 tumors differed to the G3a group with respect to the IRS of the SSTR2A (12.0 vs. 4.0; *p* < 0.001) and of the SSTR3 expression (4.0 vs. 3.0; *p* = 0.028). Significant differences between G2 and G3b tumors were seen regarding the presence of the SSTR1 (IRS: 3.0 vs. 0.5; *p* = 0.008), the SSTR2A (IRS: 12.0 vs. 4.0; *p* < 0.001) and the CXCR4 (IRS: 3.0 vs. 7.5; *p* < 0.001). G3a tumors displayed distinct differences to the G3b subgroup in the IRS of the SSTR1 (4.0 vs. 0.5; *p* < 0.001) and the CXCR4 expression (4.0 vs. 7.5; *p* = 0.006) (Table 1). As an example, in Figure 4 immunohistochemical pictures of a patient with a highly proliferative G3 neuroendocrine carcinoma are shown.

Ki-67 (IHC) correlated significantly with the IRS of the CXCR4 (r_s_: 0.39; *p* < 0.001) and with the IRS of the SSTR5 (r_s_: 0.27; *p* = 0.003) In contrast, an inverse association was seen with the IRS of the SSTR2A (r_s_: −0.50; *p* < 0.001). The IRS scores of the SSTR2A and 3 demonstrated an inverse significant interconnection with the grading of the neoplasms, whereas the IRS of the SSTR5 and of the CXCR4 presented a significant positive relation with the grading (Table 2).

**Table 2:** Spearman-rank (r_s_) and Kendall’s Tau (τ)* correlations

	Ki-67	IRS CXCR4	IRS SSTR1	IRS SSTR2A	IRS SSTR3	Grading* G1-G3
**IRS** **CXCR4**	r_s_: 0.39 *p* < 0.001	-				τ: 0.31 *p* < 0.001
**IRS** **SSTR1**	r_s_: −0.09 *p* = 0.324	r_s_: −0.17 *p* = 0.074	-			τ: −0.09 *p* = 0.257
**IRS** **SSTR2A**	r_s_: −0.50 *p* < 0.001	r_s_: −0.08 *p* = 0.390	r_s_: 0.01 *p* = 0.941	-		τ: −0.45 *p* < 0.001
**IRS** **SSTR3**	r_s_: −0.20 *p* = 0.036	r_s_: −0.18 *p* = 0.058	r_s_: 0.25 *p* = 0.006	r_s_: 0.48 *p* < 0.001	-	τ: −0.21 *p* = 0.008
**IRS** **SSTR5**	r_s_: 0.27 *p* = 0.003	r_s_: 0.04 *p* = 0.690	r_s_: 0.16 *p* = 0.085	r_s_: 0.17 *p* = 0.061	r_s_: 0.36 *p* < 0.001	τ: 0.19 *p* = 0.016

**Figure 5:**
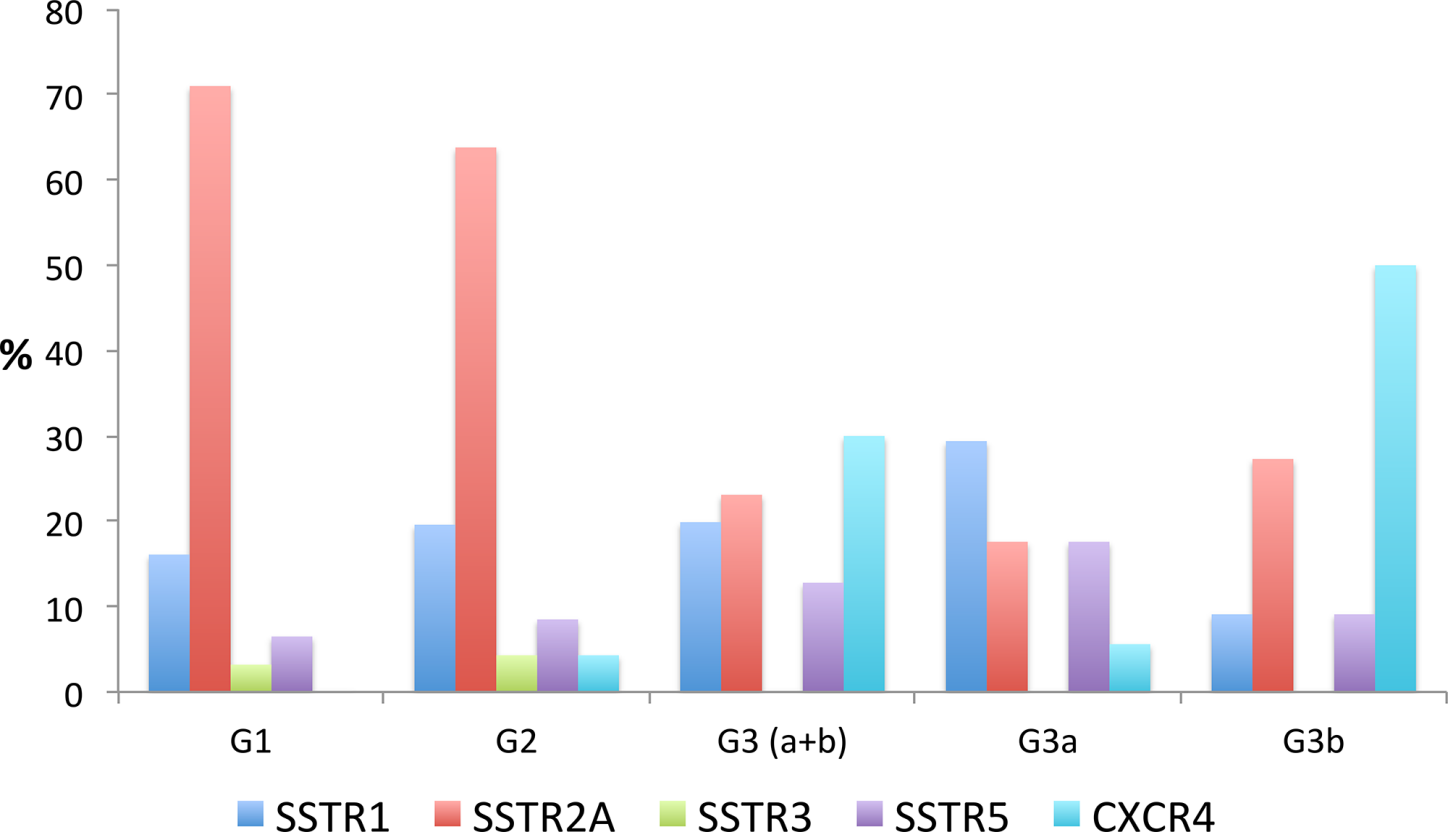
Percentage of positive cases with a strong SSTR subtype and CXCR4 expression (IRS > 8 points) within the different tumor groups (G1 – G3)

The SSTR subtype and CXCR4 expression of all positively stained specimens is shown for different staining levels in Table 3 (IRS > 2 and < 5 = weak staining; IRS ≥ 5 and ≤ 8 = moderate staining; IRS > 8 = strong staining; Figure 5).

**Table 3:** Different immunohistochemical expression levels of SSTR2A, 5 and CXCR4 (number of positive cases/total cases)

**(IRS > 2 and < 5 points)**	**G1**	**G2**	**G3**	
			**G3a**	**G3b**
**CXCR4**	11/31 (35.5%)	26/47 (55.3%)	13/18 (72.2%)	19/22 (86.4%)
**SSTR2A**	31/31 (100%)	45/47 (95.7%)	12/17 (70.6%)	14/22 (63.6%)
**SSTR5**	20/31 (64.5%)	42/47 (89.4%)	13/17 (76.5%)	20/22 (90.9%)
**(IRS ≥ 5 and ≤ 8)**	**G1**	**G2**	**G3**	
			**G3a**	**G3b**
**CXCR4**	7/31 (20.0%)	10/47 (21.3%)	5/18 (27.8%)	15/22 (68.2%)
**SSTR2A**	28/31 (90.3%)	43/47 (91.5%)	7/17 (41.2%)	10/22 (45.5%)
**SSTR5**	10/31 (32.3%)	25/47 (53.2%)	10/17 (58.8%)	11/22 (50.0%)
**(IRS > 8)**	**G1**	**G2**	**G3**	
			**G3a**	**G3b**
**CXCR4**	0/31 (0.0%)	2/47 (4.3%)	1/18 (5.6%)	11/22 (50.0%)
**SSTR2A**	22/31 (71.0%)	30/47 (63.8%)	3/17 (17.6%)	6/22 (27.3%)
**SSTR5**	2/31 (6.5%)	4/47 (8.5%)	3/17 (17.6%)	2/22 (9.1%)

### Survival data

Between the Ki-67 values (as evaluated by immunohistochemistry) or the IRS of the CXCR4, respectively, and overall survival (OS) a significant inverse correlation was observed (r_s_: −0.46; *p* < 0.001; r_s_: −0.26; *p* = 0.042). The SSTR2A expression exhibited a non-significant positive association with OS (r_s_: 0.21; *p* = 0.110).

**Figure 6:**
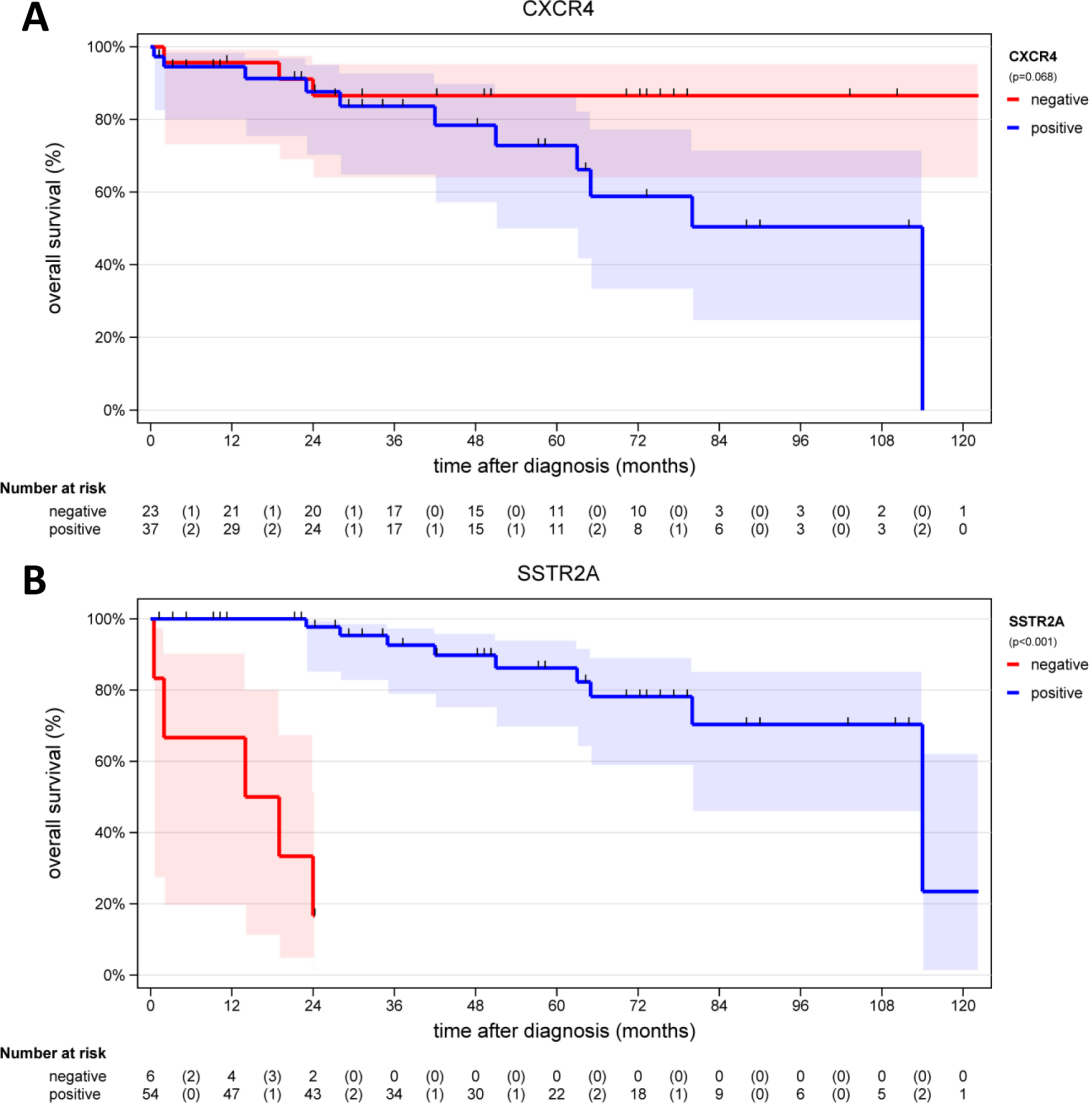
**A.** Overall survival of patients with a negative CXCR4 expression (IRS ≤ 2, *n* = 23) and of patients with a positive CXCR4 expression (IRS > 2, *n* = 37) **B.** Overall survival of patients with a negative SSTR2A expression (*n* = 6) and of patients with a positive SSTR2A expression (*n* = 54).

Patients with no CXCR4 expression (IRS ≤ 2, *n* = 23; 3 reported deaths) displayed an median OS of 50.0 months [CI: 43.6 – 75.7], whereas patients with a positive CXCR4 expression (IRS > 2, *n* = 37; 12 reported deaths) presented a distinctly lower median OS of 34.0 months [CI: 31.6 – 54.1] (Log rank *p* = 0.068 (Figure 6A). The mortality rate after 60 months was 3 vs. 7 events (chi-square *p* = 0.206) and for 114 months 3 vs. 12 events (chi-square *p* = 0.020).

Out of 60 patients 6 (10%) had an IRS of the SSTR2A ≤ 2, which was set as non-existent expression, whereas 54 of 60 patients (90%) exhibited an IRS > 2; data of 4 patients were not available. Within the group of patients displaying an IRS of the SSTR2A ≤ 2, five events of death (5/6; 83.3%) were observed and in the group with a positive SSTR2A expression one event was seen in the same time period of 24 months (1/54; 1.9%) (chi-square *p* = 0.102).

A mean OS of 13.9 ± 4.3 [CI: 2.9–24.9] months (median 16.5) was observed for the patients with no SSTR2A expression (*n* = 6), and a mean OS of 53.8 ± 4.7 [CI: 44.3–63.3] months (median 49.5) was reported for the group of patients with a positive SSTR2A expression (*n* = 54; Log rank *p* < 0.001; Figure 6B).

## DISCUSSION

In the present study, we evaluated the co-expression of different SSTR (SSTR1, 2A, 3, 5) with CXCR4 in gastroenteropancreatic neuroendocrine neoplasms (G1-G3), comprising a total of 121 samples from 64 patients, the composition of which was comparable to previous studies, which reported the small intestine and the pancreas to represent the most frequent origins of NEN [18, 19].

While CXCR4 expression has only been described for a limited number of neuroendocrine neoplasms [20, 21], more comprehensive data are available for SSTR. For example, Pisarek et al. described SSTR1 and SSTR5 as the dominant SSTR subtypes in neuroendocrine tumors [22]. In that study, the authors investigated an assortment of neuroendocrine neoplasms originating from the intestinal and the bronchopulmonary tract. However, as we previously showed, the SSTR status of intestinal and bronchopulmonary NEN is quite different and thus the results cannot be thrown together [23–25]. Here, we show that SSTR2A is overexpressed mainly in G1 and G2 tumors (with a presence in 96–100% of all cases), but that it can also be found in G3 specimens, although with lower frequency (64–71%). Our results are completely in accordance with the data of Mizutani et al., who demonstrated significant differences in SSTR2A expression levels between G1/G2 and G3 neoplasms [26]. Even when only considering cases with strong SSTR2A staining (IRS >8 points), 64–71% of the G1 and G2 tumor samples were positive for SSTR2A. This finding underlines the predominance, and thus the importance, of the SSTR2A in G1 and G2 neoplasms. In our study, 44% of the G3 specimens (17 out of 39 cases) had moderate SSTR2A staining (IRS >5 points), but only 18–27% of the G3 samples displayed strong SSTR2A staining (IRS >8 points). Recently, Zamora et al. and Okuwaki et al. reported about a similar incidence of SSTR2A expression in poorly differentiated neuroendocrine carcinomas [27, 28]. However, these studies only considered SSTR2A positivity without taking its level of expression into account. Our results are in contrast to the data of Mizutani et al., who detected SSTR2A expression in 95% and 62% of the neuroendocrine carcinoma specimens at the mRNA and protein levels, respectively [26]. This discrepancy may be due to the differences in tumor origin; most of the G3 carcinomas evaluated by Mizutani et al. originated from the lung, breast, and prostate, while our G3 specimens were derived from the intestinal tract only. Furthermore, different sensitivities of the detection systems used have to be considered [26]. In contrast, the predominance of SSTR2A expression in G1 and G2 neuroendocrine tumors, which was found in our investigation, has been observed and verified by many other studies, with an incidence of 84–100% [27, 29, 30].

In addition to SSTR2A, we also observed SSTR1 expression in the G1-G3 samples, but it was only expressed at low levels (Figure 1). Surprisingly, there was a striking difference in SSTR1 expression within the G3 group, between the G3a and G3b subtypes. Similarly, Kulaksiz et al. [30] reported low SSTR1 expression, as detected by immunohistochemistry, in 37% of the GEP-NEN. Zamora et al. also demonstrated a gradual decline in SSTR1 expression of well- and poorly differentiated neuroendocrine tumors, with a frequency of 46% and 25% of the cases, respectively [27].

SSTR3 expression has been reported by Lupp et al. in 84% and by Mizutani et al. in 52–55% of the neuroendocrine tumors of the intestine [26, 31], Other studies report incidences of about 26–71% [27, 30]. Therefore, our finding that SSTR3 is expressed in 53–79% of the samples is comparable to previous findings.

In the present investigation, SSTR5 was most notably expressed in G3 specimens, displaying up to a moderate level of expression. These data are in accordance with the results of previous studies by Papotti et al., Kulaksiz et al., and Zamora et al. [27, 30, 32], who showed a gradual increase in SSTR5 expression, ranging from 55–83% in G1 to G3 specimens. Therefore, a positive correlation between the proliferation index Ki-67 and SSTR5 expression level was to be expected.

Corleto et al. showed that neither SSTR2A expression, SSTR5 expression, nor low Ki-67 levels (<2%) alone were correlated with increased survival. However, increased survival was observed when these three factors occurred simultaneously [33]. Our data, in contrast, revealed a significant inverse relationship between the Ki-67 level and OS, as also shown by other studies [34, 35]. SSTR2A was mainly expressed in G1 and G2 tumors and had an inverse relationship with tumor grade. Of all the patients investigated in the present study (G1 - G3), 90% displayed a positive staining for SSTR2A, which was associated with significantly longer cumulative survival as compared to patients with a negligible SSTR2A expression (IRS ≤ 2). However, this result should be verified, because in our investigation only 6 cases (10% of the patients) were negative for SSTR2A. Recently, Okuwaki et al. demonstrated that SSTR2A negativity was associated with poor outcomes in patients with pancreatic neuroendocrine tumors [28]. This observation may be explained by the fact that many more treatment options are available if the tumors have sufficient SSTR2A expression.

To the best of our knowledge, there are no data available at present on the co-expression of SSTR and CXCR4 in neuroendocrine neoplasms. For that reason, we cannot compare our data to other studies. As expected, we found a strong association between the Ki-67 index and CXCR4 expression, and an inverse relationship between the Ki-67 index and strength of SSTR2A staining. Similarly, Papotti et al. demonstrated a decline in the SSTR2A expression level in tumors with a higher proliferation rate (grading) and in advanced tumor stages [32].

The SSTR subtype expression serves as the basis for therapy and the molecular imaging of neuroendocrine tumors. Previous studies have proven their efficacy in imaging and the correlation of the in-situ positron emission tomography (PET) uptake parameters with the SSTR2A expression level of the tumors [24, 36, 37]. Recently, Oksuz et al. demonstrated that in-vivo PET uptake is directly associated with the response to subsequent peptide-receptor-radionuclide-therapy (PRRT) [38]. PET uptake also correlates well with the SSTR2A expression level, as determined by immunohistochemistry [24, 36, 37]. Therefore, a strong relationship between the intensity of the SSTR2A expression and the response to PRRT treatment can be postulated. Considering the SSTR binding affinities of the peptides used at present, both for imaging and PRRT, only SSTR2A and (with much less significance) SSTR5 can be targeted with these peptides [39]. For this reason, molecular imaging or treatment with synthetic SSTR analogs is only useful for G1 and G2 tumors, which possess these receptors in an appropriate frequency and magnitude.

The current guidelines for the treatment of G3 carcinoma refer to different chemotherapy regimens (e.g., carboplatin, etoposide) with initial remission rates of up to 80% and with a duration of response of 8–11 months [40–42]. SSTR analogs are not recommended for the treatment of NEC. Overall, the treatment options are limited. Newer studies report on the possible heterogeneity of G3 neuroendocrine neoplasms with respect to their biology, and differentiate low from highly proliferative carcinomas. Additionally, different response rates to chemotherapy have been observed [5]. Our study highlights the heterogeneity of the G3 neoplasms, and describes significant differences in SSTR1 and CXCR4 expression. With regard to the prognostic value of SSTR2A, there was no detectable difference in expression within the G3 neoplasms. Surprisingly, there was still a high incidence of SSTR2A expression (SSTR2A positivity in 23% of the cases) with a strong expression intensity (IRS > 8 points) within the G3 group (Figure 5).

Many tumors express CXCR4 [43–47]. Neoplasms with high CXCR4 expression have been shown to be associated with more aggressive behavior, early metastasis, relapse, and limited survival [21]. The CXCL12/CXCR4 axis plays a crucial role in tumor development, by many different proposed mechanisms. CXCL12 stimulates the invasion and chemotactic migration of CXCR4-expressing cells, and the CXCR4/CXCL12 axis promotes angiogenesis [48, 49]. For this reason, the use of CXCR4 inhibitors is a new promising approach in cancer treatment. The first successful applications have been reported and clinical trials are ongoing [15, 50, 51]. Data on CXCR4 expression in gastroenteropancreatic neuroendocrine neoplasms, however, are limited so far [20, 21]. Deschamps et al., found that CXCR4 expression is more common in G2 than in G1 tumors, and that this expression is associated with a high rate of lymph node metastases and lower survival [21]. Our data support these findings, since we observed an increase in CXCR4 expression at the protein level from well- to poorly differentiated neoplasms and a significant correlation with tumor grade (Table 1 and 2). Additionally, we were able to demonstrate a further increase within the G3 group, from G3a to G3b neoplasms, and found a significant correlation between Ki-67 and CXCR4 expression. Similar to the findings of Deschamps et al., we also showed a significant negative correlation between CXCR4 expression and overall survival [21]. Patients negative for CXCR4 had increased survival as compared to patients positive for CXCR4 (50.0 vs. 34.0 months; Log-rank *p* = 0.068, Figure 6A).

## CONCLUSION

In the present study, the co-expression of the SSTR subtypes 1, 2A, 3 and 5 with CXCR4 was analyzed in G1–G3 neuroendocrine neoplasms. With increasing malignancy, an elevation of CXCR4 and a decrease of SSTR2A expression was seen. Interestingly, 23% of the G3 specimens had strong SSTR2A expression.

High CXCR4 expression was strongly associated with reduced overall survival, while the presence of SSTR2A expression was a strong positive prognostic factor. Because CXCR4 was strongly expressed in highly proliferative G3 carcinomas, we believe that CXCR4 is an interesting new target that needs to be validated in larger studies.

## MATERIALS AND METHODS

In the present investigation, 121 archived formalin-fixed-paraffin-embedded (FFPE) tumor samples from 64 patients were included (51 primary tumors, 70 metastases). The samples were histopathologically verified by two independent experienced pathologists as neuroendocrine tumors (G1, G2) or as neuroendocrine carcinomas (G3; Ki-67 > 20%) (Table 4). All specimens were analyzed for the expression of the SSTR-subtypes 1, 2A, 3 and 5 and of the CXCR4 by means of immunohistochemistry. All clinical data were gathered from the patient records. A positive approval from the local Ethics Committee was obtained for this retrospective study.

**Table 4:** Patient data

	G1 *N* = 18	G2 *N* = 22	G3 *N* = 24	
			G3a *N* = 10	G3b *N* = 14
***Ki-67 Index [%]***	< 2	2−20	21−49	≥ 50
***Sex [N]***				
Female	8	10	4	7
Male	10	12	6	7
***Age [years]***				
Median	58.5	58.0	62.0	62.5
Mean	58.2	57.3	60.1	62.6
Min	37.0	37.0	44.0	34.0
Max	82.0	81.0	75.0	82.0
SD	11.7	10.0	10.6	11.7
***Overall survival [months]***				
Median	49.5	64.5	23.0	14.0
Mean	57.3	63.8	35.9	13.9
Min	22.0	22.0	1.0	2.0
Max	172.0	114.0	114.0	28.0
SD	34.2	26.8	39.5	10.7

### Immunohistochemistry (IHC)

The tumor samples were embedded in paraffin blocks and sections with a thickness of 4 μm were prepared using a microtome. The sections were transferred on microscope slides and air-dried. The detection of the different SSTR subtypes and of the CXCR4 was performed using an indirect streptavidin-biotin staining method as described previously and counterstaining was done with haematoxylin [31, 52, 53].

For immunohistochemistry, only specific rabbit monoclonal antibodies (hybridoma cell culture supernatants) were employed, which are directed against the respective carboxyl-terminal tail of the different SSTR and of the CXCR4 (Epitomics, Burlingame, CA, USA; hSSTR1 [53]: UMB-7, dilution 1:30; hSSTR2A: UMB-1, dilution: 1:10 [54]; hSSTR3: UMB-5, dilution: 1:20 [31]; hSSTR5: UMB-4, dilution: 1:10 [55]; CXCR4: UMB-2, dilution: 1:100 [56]).

The analysis of the stained sections was performed by light microscopy using the immunoreactive score (IRS) according to Remmele and Stegner, comprising 12 gradations (0 – 2 ≙ negative; > 2 and < 5 ≙ weak staining; ≥ 5 and ≤ 8 ≙ moderate staining; >8 strong staining) [57, 58]. If one patient had more than one paraffin-embedded specimen, the primary tumor was preferred for analysis (46 primary tumors ≙ 46 patients). If no primary tumor of a patient was available the metastases were evaluated (18 metastases ≙ 18 patients).

### Statistics

Data were analyzed using SPSS for Windows 19.0. Due to missing normal distribution, the following parameter free statistic tests were used: Kruskall-Wallis test, Chi^2^-test, Mann-Whitney test, Kendall’s Tau (τ) and Spearman’s rank correlation analysis (r_s_). For survival analysis, the Kaplan-Meier method with a log-rank test was used. Only one representative specimen of each patient (primary tumors preferred) was considered for survival analysis (*n* = 64). All specimens (*n* = 121) were included into the Kendall’s Tau (τ) and Spearman’s rank correlation analyses. A *p*-value < 0.05 was considered statistically significant.
